# Editorial: The challenge of understanding and preventing filicide

**DOI:** 10.3389/fpsyg.2023.1159443

**Published:** 2023-04-24

**Authors:** Thea Brown, Danielle Tyson, Paula Fernandez Arias, Salmi Razali

**Affiliations:** ^1^Department of Social Work, Monash University, Caulfield, VIC, Australia; ^2^School of Humanities and Social Sciences, Deakin University, Melbourne, VIC, Australia; ^3^Department of Psychological and Behavioural Medicine, Universiti Teknologica, MARA, Seremban, Malaysia

**Keywords:** filicide, infanticide, familicide, domestic violence, child abuse, mental illness

The deaths of children, killed by a parent, stepparent, or equivalent guardian, termed filicide, are tragedies. They bring the lives of children killed to a premature end and traumatize the surviving family members and the wider community. When they happen, they prompt many questions, questions about why they have occurred and what might have been done to prevent them.

Research is grappling with these questions, trying to shed light on incidence, on the circumstances surrounding the children's deaths, and the factors associated with the victims and the perpetrators. In the first Cross Disciplinary International Filicide Research Conference, Professor Frans Koenradt (Conference Report, 2013) pointed out that filicide is a universal but not a uniform phenomenon. It varies from country to country, influenced by the culture of a country, its governmental structure, its legal structure, and its health and welfare services structure.

Even within a country, differences arise. Australian research (Brown et al., 2019) has shown differences from one state to another. For example, the state with the highest incidence, (Queensland), has almost double the incidence of the state with the lowest, (Western Australia). Furthermore, differences among the types of perpetrators have been found. While nationally biological mothers and fathers were almost equally perpetrators, in one state mothers far outnumbered fathers.

Thus, filicide needs to be investigated in every nation and even in every region. While research in one country enlightens understanding of filicide in all countries, each country has some differences in victims and perpetrators and in the causal factors associated with them. The research presented in this collection comes from four countries, Australia, Ghana, Malaysia, and Sweden. The articles highlight problems in each country and confirm the need to conduct national studies but also show cross national findings.

The article on filicide in Ghana (Abdullah et al.) might seem different in its depiction of filicide from that in other countries, but muted echoes of its findings can be found in other countries. The article investigates a type of filicide apparently unique to Ghana, the killing of a child by a parent or with the parent's compliance, because the child is seen as having been affected by spirit forces and so is liable to cause harm to the wider community. Killing the child is not murder but an easing of the child into the world awaiting it after death. Such practices, common in some rural communities, have proved difficult to prevent. Members of these communities distinguish these killing from filicide and do not wish the perpetrators to be punished or the problem to be addressed in any way. Therefore, the researchers see prevention as difficult and that responses should not focus on legal sanctions but on community education and improved health service provision.

The Swedish study (Meddeb et al.) addresses prevention but rather differently from the Ghana study. It investigates the relationship between aggressive anti-social and criminal behavior and forms of childhood disadvantage. It obtains data by administering psychological tests to patients during their residence in a high security forensic psychiatric facility. The criminal offenses the patients have committed are not identified, but by the implication of their imprisonment in a high security facility these offenses likely involve in grave physical assaults and or murder of adults and children. The study shows strong relationships between the offenders' aggressive anti-social attitudes and behavior and disinhibition and childhood trauma, particularly the removal of a child from their home and their placement outside it, parents being absent by virtue of agency intervention or for other reasons, and parental use of drugs. The study recommends more effective social work intervention both in assessing the need to remove a child from their home and in managing that removal and subsequent care. The study has clear international implications with its evidence that violent criminal behavior, typical of some male perpetrators of filicide, has been caused by childhood trauma and by the community's inadequate response to them.

The article on filicide in Malaysia (Razali et al.) aims to establish the incidence of filicide in Malaysia and to identify causal factors. It highlights the problems of obtaining data. There is no exclusive national registry on filicide in Malaysia and the reported cases are often recorded in different data bases according to police department divisions. For example, the data base of child homicide does not include cases of neonaticide or death due to illegal infant abandonment. The former cases are captured in the database of the Criminal Record Registration Division, while the latter cases are registered in the database of the Sexual, Domestic Violence and Child Abuse Investigation Division. Thus, researchers use alternate sources, including child protection case records, coroner's files, police homicide records and newspaper accounts. In this study the researchers used the police files on child homicides but point out the definitions of homicide omitted several types of filicides, thereby undermining their findings. The article touches on neonaticide, suggesting this type of filicide is particularly hard to uncover and prevent. Austrian research focusing on neonaticide agrees that this type of filicide can be hidden and be hard to prevent (Klier et al., 2019).

The article finds many factors associated with perpetrators and it supports the existence of a constellation of factors as has been identified elsewhere (Stroud, 2008; Dobash and Dobash, 2019; Johnson and Sachmann, 2019). Like other research they find that these factors, domestic violence, mental illness, drug abuse, parental separation, and a criminal history, vary according to the gender and socio-economic background of the perpetrators.

The final article (Tucci and Mitchell) takes a very different approach to all the others, but one with serious implications. It reports an ongoing study carried out repeatedly since 2003 whereby the researchers investigate Australian community attitudes and knowledge of parental violence to children. The results are alarming, for the study shows how little the community knows about violence to and abuse of children. The community has difficulty in recognizing it, in understanding it, and in taking action to protect the children. The findings also suggest community knowledge has not grown in the years since the study began but possibly deteriorated slightly. Thus, community education does not appear to have had an impact or changed attitudes to children when victims of violence nor are there adequate foundations currently for the community support needed to address violence to children.

All the articles are valuable and much needed contributions to the currently sparse knowledge on filicide and assist in creating a pathway to prevention.

## Author contributions

All authors listed have made a substantial, direct, and intellectual contribution to the work and approved it for publication.
